# Optimizing Nursing Productivity: Exploring the Role of Artificial Intelligence, Technology Integration, Competencies, and Leadership

**DOI:** 10.1155/2024/8371068

**Published:** 2024-06-26

**Authors:** Atallah Alenezi, Mohammed Hamdan Alshammari, Ibrahim Abdullatif Ibrahim

**Affiliations:** ^1^Nursing Department, College of Applied Medical Science, Shaqra University, Shaqra, Saudi Arabia; ^2^Mental Health Nursing Department, College of Nursing, University of Ha'il, Ha'il, Saudi Arabia; ^3^Nursing Administration Department, Faculty of Nursing, Mansoura University, Mansoura, Egypt

## Abstract

**Background:**

In the rapidly evolving healthcare management landscape, technology integration and artificial intelligence utilization play pivotal roles in shaping employee productivity. This research investigates these dynamics within Riyadh Province, Kingdom of Saudi Arabia, focusing on the relationships between technology integration, the use of artificial intelligence in nursing profession, nursing workforce competencies, technological leadership, and employee productivity.

**Methods:**

A quantitative approach was employed, involving 329 nurses from five hospitals in Riyadh Province. Partial Least Squares Structural Equation Modeling facilitated comprehensive analysis of direct and indirect relationships among variables.

**Results:**

Findings reveal that technology integration significantly enhances nursing productivity, while the use of artificial intelligence initially presents disruptions before yielding productivity gains. Nursing workforce competencies mediate these relationships, emphasizing the critical role of workforce readiness in harnessing technology's benefits. Surprisingly, technological leadership did not significantly moderate these effects.

**Conclusions:**

This research offers vital insights for healthcare organizations, advocating strategic technology integration and workforce development. It underscores the significance of nursing competencies in navigating technological transformations and affirms the enduring importance of leadership in guiding these changes. As healthcare evolves, these findings provide guidance for optimizing technology and artificial intelligence to enhance employee productivity and patient care.

## 1. Introduction

The continuous evolution of technology and its integration into healthcare systems have ushered in a transformative era in the field of healthcare management [1]. In the contemporary healthcare landscape, where innovation plays a pivotal role, understanding the intricate dynamics between technology, workforce competencies, leadership, and employee productivity is paramount [2, 3]. As healthcare organizations adopt technology and artificial intelligence (AI) to enhance patient care and streamline operations, understanding the implications of these advancements on employee productivity becomes crucial [1]. Technology integration, encompassing the assimilation of various technologies into healthcare processes, and the use of AI in nursing roles, representing the incorporation of AI tools into nursing roles, hold immense significance within this context [3]. Furthermore, the role of nursing workforce competencies in navigating these technological shifts and the influence of technological leadership in guiding these changes are of paramount importance [4].

Prior research has consistently emphasized the positive impact of technology integration on healthcare organizations [3, 4]. Effective technology integration can lead to improved operational efficiency and enhanced patient care outcomes [5]. Additionally, healthcare organizations that successfully integrate technology experience higher levels of employee productivity [6]. Recent studies have explored the integration of AI into nursing roles and its implications [4]. The study by Chicoine observed that while AI can optimize healthcare processes, its introduction may initially disrupt established workflows, potentially affecting productivity [4]. The AI integration requires workforce adjustments and training to mitigate any negative impact on productivity [3].

Moreover, the role of workforce competencies in healthcare settings has been extensively investigated [7]. Research has emphasized the importance of nursing competencies in effectively utilizing technology to enhance patient care [6]. Workforce competencies are critical in realizing the potential benefits of technological advancements in healthcare [8]. Additionally, the role of leadership in guiding technology adoption has been a subject of inquiry. A study by Khan highlighted the significance of transformational leadership in fostering an innovative culture within healthcare organizations [9]. Also, leadership plays a crucial role in ensuring that technology adoption aligns with organizational objective [10].

Despite the substantial body of research on each of these variables individually, there exists a notable gap in comprehensively understanding how they interact within the specific context of healthcare organizations [4]. Previous studies have often focused on isolated aspects, neglecting the holistic interplay between technology integration, the use of AI in nursing roles, nursing workforce competencies, technological leadership, and their combined impact on employee productivity [4, 6, 9].

Additionally, the specific contextual factors in Riyadh Province and the broader Kingdom of Saudi Arabia may introduce unique dynamics that warrant dedicated investigation [11, 12]. Riyadh, the capital and largest city of Saudi Arabia, boasts a diverse population and a wide array of healthcare institutions, encompassing primary care facilities and advanced hospitals. Notably, approximately 82% of private health institutions are located in Riyadh city, with the remaining 18% distributed across peripheral provinces [13]. The Kingdom of Saudi Arabia is now experiencing fast modernization and development, with a special focus on the healthcare sector. These efforts are being pushed by the Vision 2030 projects, which aim to diversify the economy and enhance healthcare services [14].

This research seeks to address these gaps by providing a comprehensive examination of these variables and their relationships, offering valuable insights for healthcare organizations navigating the evolving landscape of healthcare management. This research endeavors to shed light on these critical relationships within the context of healthcare organizations. By examining the impact of technology integration, the use of AI in nursing roles, nursing workforce competencies, and technological leadership on employee productivity, this study aims to contribute valuable insights to the healthcare management literature and offer practical implications for healthcare organizations navigating this dynamic terrain.

This study is driven by several interconnected objectives. Firstly, it aims to investigate the impact of technology integration on productivity within healthcare organizations. Secondly, it seeks to explore how the use of AI in nursing influences employee productivity in this context. Thirdly, the research aims to understand the mediating role of nursing workforce competencies in the relationships between technology integration and employee productivity, as well as the use of AI in nursing and employee productivity. Lastly, it endeavors to examine whether technological leadership moderates the relationships between technology integration and employee productivity and the use of AI in nursing and employee productivity.

Based on the identified gap in the literature and the overall objective of the study, this research aims to address the following research questions:

1. How does technology integration impact productivity within healthcare organizations?
2. What is the influence of the use of AI in nursing on employee productivity in the healthcare context?
3. What is the mediating role of nursing workforce competencies in the relationships between technology integration and employee productivity, as well as the use of AI in nursing and employee productivity?
4. Does technological leadership moderate the relationships between technology integration and employee productivity and the use of AI in nursing and employee productivity?

## 2. Literature Review and Hypothesis Development

The healthcare sector in Saudi Arabia has experienced significant growth and advancement in recent decades [11]. This shift has been marked by extensive funding for healthcare facilities, technologies, and employee training in an effort to improve healthcare delivery across the country [5]. The healthcare system in the Kingdom has undergone a transition from predominantly government-funded and government-provided to encompassing private sector involvement, fostering heightened competition and innovation [12]. Riyadh, as the capital and largest metropolis of the Kingdom, is at the forefront of advancements in medical treatment [15]. The urban area has several medical facilities, encompassing both publicly and privately funded institutions, all of which demonstrate a steadfast dedication to delivering exemplary healthcare services [15]. It is crucial for healthcare organizations to prioritize addressing issues related to nurses' productivity, as it directly impacts the quality of care delivered to patients. Recognizing the importance of improving employee productivity, healthcare organizations aim to enhance patient outcomes and ensure the highest level of care within the healthcare sector [6].

The implementation of advanced technologies has played a significant role in healthcare reform initiatives in Saudi Arabia [11]. Hospitals in Riyadh and across the Kingdom have deployed advanced information technology systems to improve patient record management, streamline administrative processes, and enhance diagnostic and therapeutic capabilities [12]. Technological integration is expected to considerably impact the delivery of healthcare services, which may have a consequential effect on healthcare workers' efficiency [16]. Furthermore, Riyadh hospitals and the healthcare industry in Saudi Arabia as a whole have given significant attention to AI in nursing. AI-driven applications such as medical diagnosis algorithms and robotic surgical aids have the potential to improve healthcare delivery [17].

The introduction of AI into nursing positions is anticipated to result in significant shifts in the nature of nursing work and its impact on overall workforce productivity [16]. The successful implementation of technology and AI in Riyadh hospitals relies heavily on the competencies of the nursing profession, another focal point of this research [15]. Healthcare personnel, including nurses, in Saudi Arabia have benefited from extensive government-funded training and development initiatives to prepare them for the impact of technological changes [18]. However, the role that these skills play in mediating the connection between tech adoption, AI utilization, and worker output is still a subject of debate [4]. Technological leadership also plays a crucial role in the current healthcare system [19, 20]. Leaders in Riyadh's hospitals, both at the managerial and clinical levels, play a critical role in advancing technological projects, fostering a spirit of innovation, and maximizing the benefits of technology and AI while minimizing their drawbacks [3, 15]. Effective leadership is essential for guiding the healthcare industry through the technological transition taking place in Riyadh, Saudi Arabia, as evidenced by the moderating influence of technological leadership on the relationship between technology integration, AI utilization, and employee productivity [17, 18].

### 2.1. Technology Integration and Employees' Productivity

Technology integration refers to the extent to which an organization incorporates advanced technologies, digital tools, and information systems into its daily operations and workflows [21]. This encompasses not only the adoption of technology but also its effective utilization to streamline processes and enhance overall organizational performance [22]. Empirical evidence from diverse sectors underscores the pivotal role of technology integration in enhancing employee productivity [23]. Employee productivity, a multifaceted concept encompassing the measurement of an employee's work output, including factors such as task completion, work quality, and efficiency, has garnered significant attention in organizational research [24]. At its core, it reflects the organization's ability to effectively utilize its human resources to achieve its goals and objectives. One prominent factor influencing employee productivity is the level of technology integration within the organization [25].

Theoretical support for the relationship between technology integration and employees' productivity can be found in the Resource-Based View (RBV) of the firm, which posits that organizations can gain sustained competitive advantages by possessing and effectively leveraging valuable and rare resources [26]. In the context of technology integration, advanced technological infrastructure becomes a valuable resource that contributes to increased productivity. By integrating technology effectively into their operations, organizations can optimize their processes, improve task efficiency, and ultimately bolster employee productivity [27]. Numerous empirical studies have corroborated the positive relationship between technology integration and employee productivity across various industries [28]. For example, firms that have successfully integrated technology into their operations have reported higher levels of employee productivity compared to those that lag in technological adoption [3]. In the healthcare sector, the adoption of health information technologies has been linked to improvements in both employee productivity and patient care outcomes [8], further emphasizing the broad applicability of this relationship.

From a theoretical standpoint, the RBV framework provides insights into how technology integration influences employee productivity by viewing technology as a valuable resource that contributes to organizational success [29]. This perspective aligns with the notion that technology, when effectively integrated, serves as a catalyst for efficiency improvement and productivity enhancement [30]. Thus, organizations that strategically leverage technology as a resource are better positioned to achieve sustainable competitive advantages and foster a culture of productivity and innovation. Therefore, we developed the following hypothesis.

**Hypothesis 1 .**

Technology integration significantly affects employees' productivity.

### 2.2. Utilization of AI and Employee Productivity

The utilization of AI in nursing occupations refers to the extent to which AI technologies, including AI-assisted diagnostics and decision support systems, are embedded within the responsibilities of nurses in healthcare settings [31]. In the context of healthcare, where precision and timeliness are paramount, the integration of AI technologies into nursing roles emerges as a significant independent variable that shapes the landscape of employee productivity [32]. Empirical investigation demonstrated that AI integration holds promise for enhancing the efficiency and productivity of healthcare personnel, particularly nurses [30].

Employee productivity, a multifaceted construct encompassing job completion, work quality, and efficiency, is a critical measure of organizational performance and effectiveness [33].

The concept of augmentation suggests that AI technologies serve to complement rather than replace human capabilities, aligning seamlessly with the notion that AI integration in nursing roles enhances employee efficiency [34]. The concept of augmentation also provides a theoretical framework for the relationship between the utilization of AI and employee productivity. Empirical evidence underscores the significant impact of AI applications on employee productivity within the healthcare sector [35]. AI-driven solutions, such as clinical decision support systems and AI-assisted diagnostics, have demonstrated potential in expediting and refining patient care processes, thereby bolstering the productivity of healthcare staff, including nurses [36]. Notably, in fields like radiology, AI algorithms have markedly improved the efficiency of radiologists' work, highlighting the positive influence of AI on productivity [37]. The theoretical underpinning of the augmentation concept further validates this hypothesis, emphasizing that the goal of AI technology is to complement human labor rather than supplant it [38]. When integrated into nursing roles, AI technologies can support healthcare professionals in tasks such as data analysis, decision making, and patient monitoring, enabling them to fulfill their responsibilities more effectively [39]. Thus, the theoretical expectation is that the utilization of AI in nursing roles will enhance their productivity by augmenting their capabilities, aligning closely with the proposed hypothesis.

**Hypothesis 2 .**

Use of AI significantly affects nurses' productivity.

### 2.3. Nursing Workforce Competencies as Mediator

Nurses' competencies encompass all aspects of nursing and include knowledge, attitude, and skills [40]. Nursing workforce competencies are pivotal in facilitating the effective utilization of technology and thereby contributing to organizational productivity [31]. Extensive empirical research has consistently highlighted the positive correlation between technology integration and employee productivity across diverse industries, underlining the significance of technological advancements in enhancing organizational performance [41]. Similarly, studies have underscored the profound impact of a highly competent nursing workforce on organizational productivity, emphasizing the critical role of competencies in driving efficiency and effectiveness [27]. However, while existing research has independently explored the relationships between technology integration, employee productivity, and the influence of competencies on productivity, there remains a dearth of empirical studies directly investigating the mediating role of nursing workforce competencies in the context of technology integration within healthcare settings [42]. Nonetheless, given the crucial role of nursing competencies in leveraging the potential of technology, it is theoretically plausible to posit that nursing workforce competencies serve as a mediating mechanism in the relationship between technology integration and employee productivity [28]. The theoretical support for this hypothesis based on the Job Demands-Resources (JD-R) model suggests that job demands and job resources influence employee outcomes, including productivity [43]. In the context of technology integration in nursing, competencies can be seen as job resources. By possessing the necessary competencies, nurses are better equipped to manage the demands of using technology in their work. These competencies act as mediators between technology integration and employee productivity, as they enable nurses to effectively navigate and utilize technology, leading to improved productivity outcomes [44].

Grounded in this theoretical framework, it is proposed that the competencies possessed by nursing professionals act as a conduit between the integration of technology and its subsequent impact on employee productivity [45]. As nurses acquire greater proficiency and knowledge in effectively utilizing technology, they are better equipped to optimize their productivity levels, thereby supporting the proposition of the mediating role of nursing workforce competencies in the context of technology integration [46]. This hypothesis underscores the intricate interplay between technology integration, nursing workforce competencies, and employee productivity, emphasizing the need for further empirical investigation to unravel the underlying mechanisms and implications for healthcare organizations.

**Hypothesis 3 .**

Nursing workforce competencies significantly mediate the relationship between technology integration and employees' productivity.

Empirical research has emerged as a strong advocate for the integration of AI within healthcare, particularly in nursing roles, as a means to bolster efficiency and productivity [39]. This body of evidence underscores the transformative potential of AI technologies in streamlining processes, optimizing decision making, and improving patient outcomes. Concurrently, a wealth of studies has consistently highlighted the positive correlation between a highly competent nursing workforce and organizational productivity [38].

The competencies possessed by nursing professionals, including clinical expertise, critical thinking skills, and technological proficiency, are acknowledged as key drivers of operational effectiveness and quality of care delivery [37]. However, despite the abundance of research in both domains, there remains a notable gap in the literature concerning the direct exploration of the mediating role of nursing workforce competencies in the context of AI utilization within nursing roles [36]. This gap presents an opportunity for theoretical exploration and empirical investigation to shed light on the intricate interplay between these variables [34].

The theoretical framework supporting the hypothesis of nursing workforce competencies as a mediator between AI utilization and employee productivity is rooted in the concept of mediation, a fundamental tenet of causal inference in social science research [30]. According to this framework, nursing workforce competencies serve as an intermediary mechanism through which the utilization of AI technologies influences employee productivity. The rationale behind this proposition lies in the premise that more competent nurses are inherently better equipped to leverage AI tools effectively within their roles [21].

Competencies related to AI, such as data interpretation, algorithm understanding, and system integration, empower nurses to harness the full potential of AI technologies to enhance their workflow efficiency, clinical decision making, and overall productivity [25]. As nursing professionals acquire and apply these competencies in their daily practice, they are theoretically poised to optimize their productivity levels, thereby bridging the gap between AI utilization and enhanced employee productivity [24]. This theoretical perspective not only provides a conceptual framework for understanding the dynamics at play but also offers practical insights for healthcare organizations seeking to leverage AI technologies to their fullest potential [22].

In summary, the hypothesis of nursing workforce competencies as a mediator between AI utilization and employee productivity represents a novel avenue for research inquiry in the field of healthcare management and nursing practice [23]. By elucidating the underlying mechanisms and pathways through which AI technologies impact productivity outcomes, this line of research has the potential to inform evidence-based strategies for workforce development, technology implementation, and organizational performance improvement within healthcare settings.

**Hypothesis 4 .**

Nursing workforce competencies significantly mediate the relationship between the use of AI and nurses' productivity.

### 2.4. Technological Leadership as Moderator

Technological leadership in healthcare refers to utilizing advanced technology as AI applications to enhance the quality of care and assist patients, healthcare professionals, and organizations in diagnosis, treatment, safety, and resource allocation [19]. Technological leadership in healthcare requires proactive and visionary leaders that actively promote digital innovation, set a good example, and adapt to changing circumstances to ensure the effective adoption of health information technology in the healthcare field [47].

Technological leadership serves as a crucial determinant of an organization's ability to effectively adopt and implement technology, thereby fostering innovation and maximizing its utilization [3]. Empirical research consistently underscores the positive relationship between technology integration and employee productivity, highlighting the importance of integrating advanced technologies into organizational workflows [26]. Additionally, studies have emphasized the pivotal role of effective leadership in shaping the outcomes of technology adoption and implementation across various industries. However, despite the recognized significance of leadership in technology-related decisions and organizational culture, limited empirical research directly examines the moderating effect of technological leadership on the relationship between technology integration and employee productivity within healthcare settings [33]. Nevertheless, it is theoretically plausible to posit that technological leadership acts as a moderator in this relationship, potentially enhancing or attenuating its strength depending on the effectiveness of leadership practices [32]. The theoretical basis for this hypothesis draws upon the concept of moderation, which suggests that technological leadership can exert an influence on the strength and direction of the relationship between technology integration and employee productivity [31].

Effective leadership practices may facilitate the successful integration and utilization of technology, thereby amplifying its positive impact on employee productivity. Conversely, ineffective leadership may impede technology integration efforts, resulting in a weaker relationship between technology adoption and productivity [41]. The theoretical underpinning of this hypothesis underscores the importance of leadership in shaping organizational processes and outcomes, particularly in the context of technology adoption and utilization [27]. By recognizing the moderating role of technological leadership, healthcare organizations can better understand and leverage leadership practices to optimize the impact of technology on employee productivity [28]. Further empirical research is warranted to empirically validate this theoretical proposition and elucidate its implications for healthcare management and leadership practices.

**Hypothesis 5 .**

Technological leadership significantly moderates the relationship between technology integration and nurses' productivity.

Empirical research has unequivocally demonstrated the potential of AI to enhance employee productivity within healthcare, particularly in nursing professions [48]. Simultaneously, studies have underscored the critical role of leadership in shaping the outcomes of technology adoption and deployment across various industries [44]. However, despite the growing body of evidence supporting the individual impacts of AI utilization and technological leadership, there exists a gap in empirical research regarding the moderating effect of technological leadership on the relationship between AI utilization in nursing roles and employee productivity within healthcare settings [45]. Nevertheless, from a theoretical standpoint, it is plausible to suggest that technological leadership acts as a moderator in this relationship, exerting influence on the strength and direction of the impact of AI utilization on employee productivity based on leadership effectiveness [46].

The theoretical foundation for this hypothesis is grounded in the concept of moderation, which posits that technological leadership can shape the relationship between the use of AI in nursing roles and employee productivity [39]. Effective leadership practices may facilitate the successful integration and utilization of AI technologies within nursing workflows, thereby enhancing employee productivity [38]. Conversely, ineffective leadership may impede AI adoption efforts, resulting in a weaker relationship between AI utilization and productivity [37]. Thus, the theoretical support for this hypothesis stems from the notion that technological leadership can moderate the relationship between AI utilization in nursing roles and employee productivity within healthcare organizations [36]. In essence, understanding the moderating role of technological leadership can provide valuable insights for healthcare organizations seeking to optimize the impact of AI technologies on employee productivity [34]. Further empirical research is needed to validate this theoretical proposition and explore its implications for leadership practices and technology implementation strategies in healthcare settings.

**Hypothesis 6 .**

Technological leadership significantly moderates the relationship between the use of AI in nursing roles and employee productivity.

**Figure 1:**
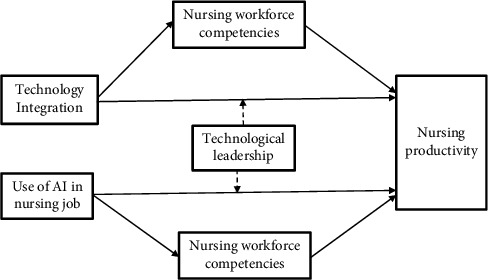
Conceptual and theoretical model of the study.

Based on the aforementioned review of literature and hypothesis development, the conceptual and theoretical model of the study was developed as shown in Figure 1.

### 2.5. Methodology

This study adopted a quantitative research design to investigate the intricate relationships among key variables in healthcare organizations located in Riyadh Province, Kingdom of Saudi Arabia. A purposive sampling method was employed to select a sample of 329 nurses, drawn from five different hospitals within Riyadh Province, ensuring representation from diverse healthcare facilities. The participants met specific inclusion criteria, namely, active involvement in clinical nursing practice within the selected hospitals.

To assess the research variables, validated measurement scales and items from previous research were adapted and customized to align with the unique context of this study. The key variables under scrutiny included technology integration, the use of AI, nursing workforce competencies, technological leadership, and productivity among nursing workforce.

The study variables were measured using adapted items from the scales of previous studies as follows: Technology integration was measured with three items [49]. The use of AI in nursing jobs was measured with eight items [50]. A three-item scale was employed for assessing nursing workforce competencies [51]. Technological leadership was measured with seven items [52]. Nursing productivity was measured on a six-item scale [53]. The participants rated their responses on a five-point Linkert scale.

Data analysis was conducted using Partial Least Squares Structural Equation Modeling (PLS-SEM), a robust statistical technique suitable for analyzing complex models, particularly in scenarios with smaller sample sizes and a focus on prediction and understanding intricate relationships. PLS-SEM allows for the examination of both direct and indirect effects among variables, making it an ideal choice for this research.

The analytical process consisted of several steps, including data preprocessing, measurement model assessment, structural model estimation, and mediation and moderation analysis. Data were cleaned, checked for outliers, and assessed for normality to ensure data validity. Missing data were managed using appropriate imputation techniques.

The measurement model was evaluated to confirm the reliability and validity of measurement scales. This involved assessments of composite reliability (CR), average variance extracted (AVE), and discriminant validity. The structural model was then estimated to explore relationships among variables, and mediation and moderation effects were analyzed to understand the roles of nursing workforce competencies and technological leadership in mediating and moderating relationships.

To ensure research validity and reliability, rigorous strategies were employed, including robust questionnaire design, adaptation of validated scales, and meticulous data analysis techniques. The quality and accuracy of the measurement model were validated through assessments CR, AVE, and discriminant validity.

Data collection was conducted through a meticulously designed structured questionnaire, tailored specifically for this study. To maximize convenience and efficiency, participants were administered the questionnaire electronically via e-mail and online survey platforms. Clear instructions were provided, emphasizing the research's purpose and the importance of providing accurate responses. Data collection was conducted over a defined period to ensure consistency and minimize potential external influences.

Ethical approval for this study was obtained from the Research Ethics Committee at the University of Hail in Saudi Arabia (Reference number: H-2023-298; dated: August 2, 2023). The study was conducted in accordance with the principles of the Declaration of Helsinki. Participants were informed of the study's aim, voluntary participation, and right to withdraw without penalty. Each individual gave informed consent before completing the questionnaire. All responses were kept strictly confidential for research purposes only, and the results did not personally identify respondents. The data collection period was carefully planned to maintain consistency and minimize potential temporal variations that could affect research outcomes. Data handling and storage adhered to data protection regulations to uphold participant confidentiality and privacy.

## 3. Results

**Figure 2:**
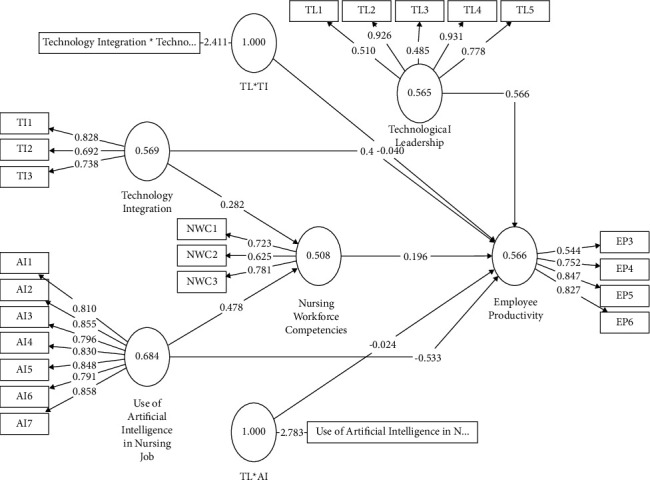
Measurement model.

Table 1 and Figure 2 show that the item loadings of utilization of AI in nursing jobs construct vary from 0.791 to 0.858, suggesting a robust association between the items and the construct. The CR rating of 0.938 above the required threshold of 0.7 suggests excellent internal consistency. The AVE score of 0.684 indicates that 68.4% of the variability in the construct is explained by the items. Cronbach's alpha coefficient of 0.923 provides additional evidence supporting the dependability of the concept.

Furthermore, the item loadings of employee productivity construct vary from 0.544 to 0.847, suggesting a reasonable correlation between the items and the construct. The CR value was 0.835 above the predetermined threshold, indicating a high level of internal consistency. The AVE of 0.566 indicates that 56.6% of the variation in the construct is explained by the components. Cronbach's alpha coefficient of 0.741 provides additional evidence for the dependability of the concept.

The item loadings of nursing workforce competencies construct vary from 0.625 to 0.781, suggesting a satisfactory correlation between the items and the construct. The CR value of 0.754 satisfies the required level, suggesting satisfactory internal consistency. The AVE score of 0.508 indicates that 50.8% of the variability in the construct can be accounted for by the components. Cronbach's alpha coefficient of 0.729 provides additional evidence of the construct's dependability. The item loadings of technology integration construct vary from 0.692 to 0.828, suggesting a reasonable correlation between the items and the construct. The CR value of 0.798 above the threshold indicates excellent internal consistency. The AVE score of 0.569 indicates that 56.9% of the variability in the construct can be explained by the items. Cronbach's alpha coefficient of 0.724 provides additional evidence for the dependability of the concept.

The item loadings of technological leadership vary from 0.510 to 0.931, suggesting a satisfactory correlation between the items and the construct. The CR value of 0.858 above the threshold indicates excellent internal consistency. The AVE score of 0.565 indicates that 56.5% of the variability in the construct is accounted for by the components. Cronbach's alpha coefficient of 0.806 provides additional evidence supporting the dependability of the concept.

Table 2 presents the Fornell–Larcker model, which assesses the discriminant validity of the study's constructs. This model examines whether each construct is more strongly correlated with its own latent variable than with other latent variables, thereby confirming their distinctiveness. The diagonal elements of the table display the square roots of the AVE values for each construct. For employee productivity, the diagonal value is 0.752, indicating that the AVE of this construct is greater than its correlations with other constructs, confirming its discriminant validity. Similarly, for nursing workforce competencies, the diagonal value is 0.712, exceeding its correlations with other constructs, supporting its distinctiveness. Technological leadership also demonstrates distinctiveness, as its diagonal value of 0.751 is higher than its correlations with other constructs. Technology integration exhibits distinctiveness with a diagonal value of 0.755, surpassing its correlations with other constructs. Finally, the use of AI in nursing job construct displays strong discriminant validity, as its diagonal value of 0.827 is greater than its correlations with other constructs. These results from the Fornell–Larcker model confirm that each construct in the study is distinct from the others, substantiating their discriminant validity and supporting the robustness of the measurement model.

Table 3 presents the results based on the Heterotrait-Monotrait (HTMT) criterion, which evaluates the discriminant validity of the study's constructs. The HTMT values indicate the extent to which constructs are more correlated with their own latent variables compared to others. For employee productivity, the table is empty, indicating that this construct does not correlate with other constructs beyond its latent variable. For nursing workforce competencies, the HTMT value is 0.741, suggesting that it has a stronger correlation with its own latent variable than with others, supporting its discriminant validity. Technological leadership exhibits a HTMT value of 0.749, indicating that it is more strongly correlated with its own latent variable than with other constructs, thus confirming its distinctiveness. Technology integration demonstrates HTMT values of 0.810, 0.711, and 0.670 with other constructs, all below the threshold of 0.85, which is indicative of discriminant validity. The use of AI in nursing job exhibits HTMT values of 0.335, 0.603, 0.874, and 0.757, all below the 0.85 threshold, indicating that this construct is distinct from others and possesses discriminant validity. These results based on the HTMT criterion affirm the discriminant validity of each construct in the study, providing confidence in the measurement model's robustness.

Table 4 displays the model fit statistics, which assess the predictive quality of the research model. The Q^2^predict value, equal to 0.612, represents the model's predictive accuracy. This value suggests that the research model has a good predictive capability, indicating that it can effectively estimate and explain the relationships among the studied variables. A root mean square error (RMSE) of 0.073 was found. The root mean square error (RMSE) is a measure of how well a model fits the data, and a smaller RMSE suggests that the model's predictions are close to the observed values. The mean absolute error (MAE) value is also provided (0.084). The mean absolute error (MAE) quantifies how off estimates are, on average. Higher predictive accuracy is indicated by a smaller MAE, which indicates that the model's predictions are, on average, quite close to the actual data. Taken together, these model fit statistics indicate that the research model is a good fit for the data and may be used to make informed inferences about the relationships between the variables of interest.

Table 5 presents the R-square values for the study's variables, which indicate the proportion of variance in the dependent variable explained by the independent variables. For employee productivity, the R-square value is 0.636. This indicates that approximately 63.6% of the variance in employee productivity can be explained by the independent variables in the model, suggesting a strong explanatory power. For nursing workforce competencies, the *R*-square value is 0.467. This suggests that the model's explanatory power is moderate, as the independent variables account for about 46.7% of the variance in nursing workforce competencies. These *R*-square values assist in evaluating the overall explanatory power of the model by showing how well the independent variables in the study explain the variability in the relevant dependent variables.

Table 6 provides valuable insights into the *F*-square values, which illuminate the proportion of variance in the dependent variable explained by the independent variables within the study. In the context of employee productivity, these F-square values demonstrate the contributions of each independent variable: technological leadership emerges as a significant factor, explaining approximately 35.1% of the variance in employee productivity. Technology integration also plays a substantial role, contributing around 26.4% to the explanation of employee productivity. Additionally, the use of AI in nursing profession exhibits notable influence, contributing approximately 26.5% to the explanation of employee productivity. Nursing workforce competencies contribute about 5.3% to the explanation of the variance in employee productivity. For nursing workforce competencies, it is noteworthy that technological leadership contributes about 9.7% to the explanation of the variance in nursing workforce competencies. Technology integration contributes around 2.64% to the explanation of nursing workforce competencies, and it also contributes about 5.3% to its own variance. These F-square values collectively shed light on the explanatory power of the independent variables in relation to the dependent variables.

**Figure 3:**
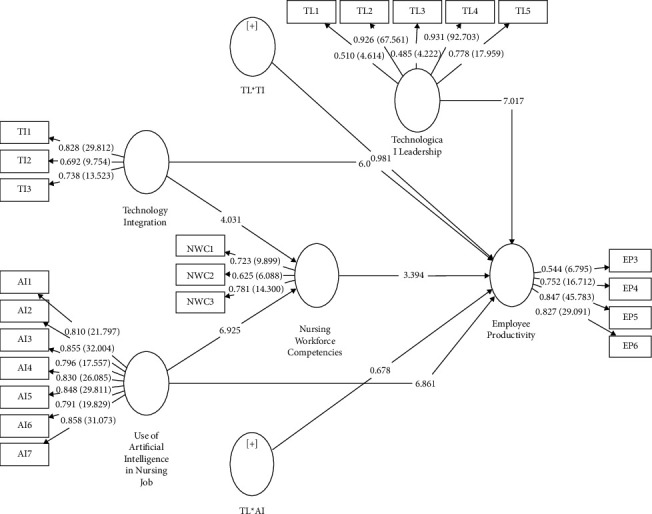
Structural model results.

Table 7 presents the results of the path analysis, which elucidate the relationships between the variables in the research model. First, the path from technology integration to employee productivity demonstrates a positive relationship with a path coefficient of 0.437. This finding indicates that an increase in technology integration is associated with higher employee productivity (see Figure 3). The T-statistic of 6.093 is statistically significant (*p* < 0.001), underlining the robustness of this relationship. Similarly, the path from the use of AI in nursing jobs to employee productivity shows a negative relationship with a path coefficient of −0.533. This implies that a greater use of AI in nursing jobs is linked to lower employee productivity. This relationship is statistically significant with a *T*-statistic of 6.861 (*p*<0.001).

Moreover, the indirect path from technology integration to nursing workforce competencies and subsequently to employee productivity reveals a positive relationship with a path coefficient of 0.055. This indicates that as technology integration increases, it positively affects nursing workforce competencies, which, in turn, enhances employee productivity. This path is statistically significant, with a *T*-statistic of 2.354 (*p* = 0.009). Similarly, the indirect path from the use of AI in nursing jobs to nursing workforce competencies and then to employee productivity shows a positive relationship with a path coefficient of 0.094. This suggests that an increased use of AI in nursing jobs positively influences nursing workforce competencies, subsequently leading to higher employee productivity. This path is also statistically significant, with a T-statistic of 3.214 (*p* = 0.001). Regarding moderation effects, the interaction terms (TLAI and TLTI) did not yield statistically significant relationships with employee productivity. The path coefficients for TLAI and TLTI are −0.024 and −0.040, respectively, with T-statistics of 0.678 (*p* = 0.249) and 0.981 (*p* = 0.164), suggesting that technological leadership does not significantly moderate the relationship between the use of AI in nursing duties or technology integration and employee productivity. These path analysis results provide a comprehensive understanding of how the variables in the study are interrelated and their respective impacts on employee productivity within the context of the research model (see Table 7).

## 4. Discussion

The purpose of the research was to examine the correlations between technological leadership, employee productivity, nursing workforce competencies, AI usage in the nursing jobs, and technological integration among nursing staff in the healthcare organizations at Riyadh Province, Saudi Arabia. A positive and statistically significant association between technology integration and employee productivity was indicated by the data, which verified Hypothesis 1. This finding aligns with previous research suggesting that effective integration of technology can enhance employee productivity [17]. Hypothesis 2 was also supported by the results, revealing a negative and statistically significant relationship between the use of AI in nursing job and employee productivity. This is consistent with prior studies, which suggest that the introduction of AI in healthcare settings may necessitate workforce adjustments and potentially lead to initial decreases in productivity [3]. The mediation effect proposed in Hypothesis 3 was substantiated by the data. It was found that nursing workforce competencies significantly mediate the relationship between technology integration and employee productivity. This result emphasizes the crucial role of nursing competencies in harnessing the benefits of technology, aligning with existing literature [24]. Hypothesis 4 was also supported, with nursing workforce competencies mediating the relationship between the use of AI in nursing duties and employee productivity. This underscores the importance of competence development among nurses to maximize the positive impact of AI technologies, in line with prior studies [3].

Hypothesis 5 did not receive empirical support, as technological leadership was not found to significantly moderate the relationship between technology integration and employee productivity. This finding contrasts with some existing research [48] and suggests that within the healthcare context studied, leadership may not play a significant moderating role in this specific relationship. Similarly, Hypothesis 6 was not supported by the data. Technological leadership was not found to significantly moderate the relationship between the use of AI in nursing tasks and employee productivity. This outcome may indicate that, within the healthcare context studied, leadership did not exert a notable moderating influence on this specific relationship.

The findings of this research provide valuable insights into the intricate dynamics between technology, workforce competencies, leadership, and productivity in healthcare organizations in Riyadh Province, Kingdom of Saudi Arabia. They underscore the need for healthcare institutions to carefully manage the integration of technology and the introduction of AI into nursing roles to optimize employee productivity. The positive relationship between technology integration and employee productivity highlights the potential benefits of effectively adopting and integrating advanced technologies in healthcare settings. This finding supports the idea that technology can enhance operational efficiencies and ultimately lead to improved productivity, which is consistent with previous literature [24]. Conversely, the negative relationship between the use of AI in nursing duties and employee productivity suggests that while AI technologies offer significant advantages in healthcare, their implementation can initially disrupt established workflows and processes. This finding resonates with research indicating that the introduction of AI may require adjustments and training to mitigate any negative impact on productivity [48].

The mediating role of nursing workforce competencies in both relationships emphasizes the importance of equipping healthcare professionals, particularly nurses, with the necessary skills and knowledge to effectively use technology and AI tools. This finding aligns with existing literature emphasizing the role of workforce competencies in realizing the potential benefits of technological advancements in healthcare [54]. Surprisingly, the study did not find significant moderation effects of technological leadership in either relationship. This result suggests that, in the specific healthcare context studied, leadership may not exert a substantial influence on the relationship between technology adoption and employee productivity. However, it is important to note that leadership remains a critical component of successful technology implementation in healthcare organizations [54].

### 4.1. Limitations and Future Research Directions

While this research contributes valuable insights to the field of healthcare management and technology adoption, it is important to acknowledge its limitations. Firstly, the study was conducted in the specific context of healthcare organizations in Riyadh Province, Kingdom of Saudi Arabia. The findings may not be directly transferable to diverse cultural, organizational, or regional settings. Future research should explore the generalizability of these findings across diverse healthcare contexts. Secondly, the research primarily relied on quantitative data, and the results are based on self-reported measures. While this approach provides valuable quantitative insights, it may not capture the full spectrum of qualitative nuances and contextual factors that influence technology adoption and employee productivity in healthcare settings. Future studies could incorporate qualitative methods to provide a richer understanding of these dynamics. Thirdly, this research focused on a selected set of variables, primarily technology integration, the use of AI, nursing workforce competencies, and technological leadership. While these variables are critical within the healthcare context, there may be additional factors, such as organizational culture, resource availability, and patient demographics, that influence the relationships explored in this study. Future research should consider a broader range of variables to provide a more comprehensive analysis.

Building on the insights gained from this research, several avenues for future investigation emerge. Firstly, further exploration of the role of leadership in technology adoption and its impact on employee productivity is warranted. The qualitative studies can delve into the leadership styles and practices that effectively facilitate technology integration in healthcare organizations. Secondly, as technology and AI continue to evolve rapidly, ongoing research is needed to assess their long-term effects on employee productivity and patient outcomes. Longitudinal studies can provide valuable insights into the sustainability of productivity gains and potential adjustments required over time. Thirdly, cross-cultural research can shed light on how cultural factors influence the relationships examined in this study. Comparative analyses across different healthcare systems and regions can enhance our understanding of the generalizability of these findings and the role of cultural contexts. Lastly, investigations into the impact of specific AI applications and technologies on nursing roles and patient care outcomes could offer specialized insights. Research could focus on the effectiveness of AI tools in tasks such as diagnostics, treatment planning, and patient monitoring, providing a more granular understanding of their effects.

In summary, this research lays the foundation for future studies to explore, refine, and expand upon the relationships between technology adoption, workforce competencies, leadership, and employee productivity in healthcare settings. Addressing these limitations and pursuing these research directions will contribute to a more nuanced and comprehensive understanding of technology-driven transformations in healthcare management.

### 4.2. Implications of the Study

#### 4.2.1. Theoretical Implications

This research has several theoretical implications that contribute to the existing body of knowledge in healthcare management and technology adoption. Firstly, it reinforces the importance of considering the mediating role of nursing workforce competencies when examining the impact of technology integration and the use of AI on employee productivity. This mediation framework provides a more comprehensive understanding of the complex relationships within healthcare organizations. Secondly, the findings challenge the assumption that technological leadership invariably moderates the relationship between technology adoption and employee productivity. This suggests that the influence of leadership may vary depending on contextual factors and organizational dynamics. It prompts further exploration of the nuanced role of leadership in technology-driven healthcare environments.

#### 4.2.2. Practical Implications

Based on the study's findings, healthcare organizations should develop comprehensive training programs to enhance nurses' competencies in technology utilization. This can include specific training on various technologies, such as Electronic Health Record (EHR) systems, telemedicine platforms, and remote monitoring devices. By equipping nurses with the necessary skills and knowledge, they will be better prepared to effectively leverage technology in their daily practice.

While the study did not find significant moderation effects of technological leadership, it is still an essential component of successful technology implementation. Therefore, healthcare leaders should prioritize the development of a culture that encourages innovation and embraces technological advancements. Creating platforms for nurses to share insights and ideas on utilizing technology to improve processes can foster collaboration between nurses and technology specialists. Additionally, establishing innovation committees or task forces can help drive the adoption of innovative solutions.

Continuous monitoring and evaluation of technology integration's impact on nurse productivity and job satisfaction is crucial. Regular assessments, surveys, or feedback mechanisms can be utilized for this purpose. By collecting and analyzing data on the effectiveness of technology implementation, healthcare organizations can identify areas for improvement and make necessary adjustments to optimize outcomes.

To facilitate a smooth transition to technology integration, healthcare organizations should provide the necessary resources and support systems. This includes offering technical support, providing access to training materials and resources, and ensuring that nurses have sufficient time and support to adapt to new technologies. Establishing regular communication and feedback channels will address any concerns or challenges that may arise during the implementation process.

Healthcare organizations should also develop workforce adjustment strategies. This can involve reevaluating job roles and responsibilities, considering workload redistribution, or providing additional training and support to help nurses adapt to AI technologies. Proactive measures to address potential challenges can mitigate the negative impact on productivity and promote job satisfaction. By implementing these practical recommendations, healthcare organizations can enhance nurse productivity while increasing job satisfaction. This, in turn, can lead to improved patient outcomes and overall organizational performance.

## 5. Conclusions

In conclusion, this research has provided valuable insights into the complex dynamics between technology integration, the use of AI in nursing jobs, nursing workforce competencies, technological leadership, and employee productivity within healthcare organizations in Riyadh Province, Kingdom of Saudi Arabia. The study confirmed that effective technology integration positively influences employee productivity, while the use of AI, while beneficial in the long term, may initially pose productivity challenges. Furthermore, the mediating role of nursing workforce competencies emphasizes the critical need for ongoing training and skill development to harness the full potential of technology and AI in healthcare settings. Although this study did not find significant moderating effects of technological leadership, it is essential to recognize the enduring importance of leadership in guiding technological advancements and change management in healthcare organizations. Leaders must play a pivotal role in fostering a culture of innovation and ensuring that technology adoption aligns with organizational goals. The findings underscore the significance of a well-prepared and adaptable healthcare workforce, capable of embracing and effectively utilizing technological advancements to enhance patient care and overall organizational performance. Healthcare organizations should prioritize workforce development programs and training initiatives to bridge competency gaps and facilitate the successful integration of technology. In summary, this research contributes to the understanding of how technology, AI, competencies, and leadership intersect in the healthcare sector. It provides healthcare administrators and policymakers in Riyadh Province, Kingdom of Saudi Arabia, with valuable insights to inform strategic decisions and investments in technology, workforce development, and leadership practices. As the healthcare landscape continues to evolve, these insights will be instrumental in ensuring that organizations can maximize the benefits of technological advancements while maintaining and improving employee productivity and patient care.

## Figures and Tables

**Table 1:** Reliability and validity statistics of the study variables.

Construct	Items	Item loading	CR	AVE	Cronbach's alpha
Use of AI in nursing jobs	AI1	0.810	0.938	0.684	0.923
	AI2	0.855			
	AI3	0.796			
	AI4	0.830			
	AI5	0.848			
	AI6	0.791			
	AI7	0.858			

Employee productivity	EP3	0.544	0.835	0.566	0.741
	EP4	0.752			
	EP5	0.847			
	EP6	0.827			

Nursing workforce competencies	NWC1	0.723	0.754	0.508	0.729
	NWC2	0.625			
	NWC3	0.781			

Technology integration	TI1	0.828	0.798	0.569	0.724
	TI2	0.692			
	TI3	0.738			

Technological leadership	TL1	0.510	0.858	0.565	0.806
	TL2	0.926			
	TL3	0.585			
	TL4	0.931			
	TL5	0.778			

**Table 2:** Fornell–Larcker model.

	1	2	3	4	5
Employee productivity	0.752				
Nursing workforce competencies	0.481	0.712			
Technological leadership	0.646	0.520	0.751		
Technology integration	0.560	0.565	0.411	0.755	
Use of AI in nursing job	0.291	0.645	0.584	0.593	0.827

**Table 3:** HTMT criterion.

	1	2	3	4	5
Employee productivity					
Nursing workforce competencies	0.741				
Technological leadership	0.693	0.749			
Technology integration	0.810	0.711	0.670		
Use of AI in nursing job	0.335	0.603	0.874	0.757	

**Table 4:** Model fit.

Q^2^predict	RMSE	MAE
0.612	0.073	0.084

**Table 5:** *R*-square.

Variable	*R*-square
Employee productivity	0.636
Nursing workforce competencies	0.467

**Table 6:** *F*-square.

The study variables	Employee productivity	Nursing workforce competencies
Nursing workforce competencies	0.053	
Technological leadership	0.351	
Technology integration	0.264	0.097
Use of AI in nursing profession	0.265	0.278

**Table 7:** Path analysis.

	Original sample	Standard deviation	*T* statistics	*P* values
Technology integration ≥ employee productivity	0.437	0.072	6.093	≤0.001
Use of AI in nursing ≥ employee productivity	−0.533	0.078	6.861	≤0.001
Technology integration ≥ nursing workforce competencies ≥ employee productivity	0.055	0.023	2.354	0.009
Use of AI in nursing ≥ nursing workforce competencies ≥ employee productivity	0.094	0.029	3.214	0.001
Technological leadership *∗* use of AI in nursing ≥ employee productivity	−0.024	0.036	0.678	0.249
Technological leadership *∗* technology integration ≥ employee productivity	−0.040	0.041	0.981	0.164

## Data Availability

The datasets used and/or analyzed during the current study are available from the corresponding author on reasonable request.

## References

[1] Dimitriadou M., Merkouris A., Charalambous A., Lemonidou C., Papastavrou E. (2021). The knowledge about patient safety among undergraduate nurse students in Cyprus and Greece: a comparative study. *BMC Nursing*.

[2] Karneli O. (2023). The role of adhocratic leadership in facing the changing business environment. *Journal of Contemporary Administration and Management (ADMAN)*.

[3] Shaikh F., Afshan G., Anwar R. S., Abbas Z., Chana K. A. (2023). Analyzing the impact of artificial intelligence on employee productivity: the mediating effect of knowledge sharing and well-being. *Asia Pacific Journal of Human Resources*.

[4] Chicoine G., Côté J., Pepin J., Boyer L., Rouleau G., Jutras-Aswad D. (2022). Experiences and perceptions of nurses participating in an interprofessional, videoconference-based educational programme on concurrent mental health and substance use disorders: a qualitative study. *BMC Nursing*.

[5] Tseng L. P., Hou T. H., Huang L. P., Ou Y. K. (2021). Effectiveness of applying clinical simulation scenarios and integrating information technology in medical-surgical nursing and critical nursing courses. *BMC Nursing*.

[6] Zhong H., Zhou L., Liao S. (2022). Effects of a fixed nurse team in the orthopaedic surgery operating room on work efficiency and patient outcomes: a propensity score-matched historically controlled study. *BMC Nursing*.

[7] Dijkman B. L., Hirjaba M., Wang W. (2022). Developing a competence framework for gerontological nursing in China: a two-phase research design including a needs analysis and verification study. *BMC Nursing*.

[8] Ur Rehman K., Anwar R. S., Antohi V. M., Ali U., Fortea C., Laura Zlati M. (2024). Driving frugal innovation in SMEs: how sustainable leadership, knowledge sources and information credibility make a difference. *Front Sociol*.

[9] Khan N. A., Khan A. N. (2019). What followers are saying about transformational leaders fostering employee innovation via organisational learning, knowledge sharing and social media use in public organisations?. *Government Information Quarterly*.

[10] Farghaly Abdelaliem S. M., Abou Zeid M. A. G. (2023). The relationship between toxic leadership and organizational performance: the mediating effect of nurses’ silence. *BMC Nursing*.

[11] Almujadidi B., Adams A., Alquaiz A., Van Gurp G., Schuster T., Andermann A. (2022). Exploring social determinants of health in a Saudi Arabian primary health care setting: the need for a multidisciplinary approach. *International Journal for Equity in Health*.

[12] Alarabi S., Alasmari F. (2023). Challenges and opportunities for small cities in the Kingdom of Saudi Arabia: a study of expert perceptions. *Sustainability*.

[13] Alshahrani H., Matrafi N., qahtani N., Taliby R., Hassanein M., Rowilly I. (2023). Mapping the private healthcare sector in Riyadh region: size, services, and alignment with the Saudi Ministry of Health priorities. *Mater Sociomed*.

[14] Alasiri A. A., Mohammed V. (2022). Healthcare transformation in Saudi Arabia: an overview since the launch of vision 2030. *Health Services Insights*.

[15] Tlili I., Alkanhal T. A., Othman M., Dara R. N., Shafee A. (2020). Water management and desalination in KSA view 2030: case study of solar humidification and dehumidification system. *Journal of Thermal Analysis and Calorimetry*.

[16] Lee S. M., Lee D. H. (2021). Opportunities and challenges for contactless healthcare services in the post-COVID-19 Era. *Technological Forecasting and Social Change*.

[17] Tariq A., Gill A. Y., Hussain H. K. (2023). Evaluating the potential of artificial intelligence in orthopedic surgery for value-based healthcare. *International Journal of Multidisciplinary Sciences and Arts*.

[18] Sajjad R., Qureshi M. O. (2020). An assessment of the healthcare services in the Kingdom of Saudi Arabia: an analysis of the old, current, and future systems. *International Journal of Healthcare Management*.

[19] Petersson L., Svedberg P., Nygren J. M., Larsson I. (2023). Healthcare leaders’ perceptions of the usefulness of AI applications in clinical work: a qualitative study. *Studies in Health Technology and Informatics*.

[20] Osipov V. S., Skryl T. V. (2021). Impact of digital technologies on the efficiency of healthcare delivery. *IoT in Healthcare and Ambient Assisted Living*.

[21] Singh N., Jain M., Kamal M. M., Bodhi R., Gupta B. (2024). Technological paradoxes and artificial intelligence implementation in healthcare. An application of paradox theory. *Technological Forecasting and Social Change*.

[22] Jaiswal A., Arun C. J., Varma A. (2022). Rebooting employees: upskilling for artificial intelligence in multinational corporations. *International Journal of Human Resource Management*.

[23] Rožman M., Oreški D., Tominc P. (2022). Integrating artificial intelligence into a talent management model to increase the work engagement and performance of enterprises. *Frontiers in Psychology*.

[24] Al Naqbi H., Bahroun Z., Ahmed V. (2024). Enhancing work productivity through generative artificial intelligence: a comprehensive literature review. *Sustainability*.

[25] Wachter R. M., Brynjolfsson E. (2024). Will generative artificial intelligence deliver on its promise in health care?. *JAMA*.

[26] Usman M. (2022). Synergy between professional human resources and artificial intelligence: a review of the consequences of technological innovation. *International Journal of Innovative Business Strategies*.

[27] Zahlan A., Ranjan R. P., Hayes D. (2023). Artificial intelligence innovation in healthcare: literature review, exploratory analysis, and future research. *Technology in Society*.

[28] Chowdhury S., Dey P., Joel-Edgar S. (2023). Unlocking the value of artificial intelligence in human resource management through AI capability framework. *Human Resource Management Review*.

[29] Zaman U., Nawaz S., Anjam M., Anwar R. S., Siddique M. S. (2021). Human resource diversity management (HRDM) practices as a coping mechanism for xenophobia at transnational workplace: a case of a multi-billion-dollar economic corridor. *Cogent Business and Management*.

[30] Lingam M. S., Vanishree J. (2024). Leadership in implementing artificial intelligence (AI) for Strategic purposes. *International Development Planning Review*.

[31] Vrontis D., Christofi M., Pereira V., Tarba S., Makrides A., Trichina E. (2022). Artificial intelligence, robotics, advanced technologies and human resource management: a systematic review. *International Journal of Human Resource Management*.

[32] Arslan A., Cooper C., Khan Z., Golgeci I., Ali I. (2022). Artificial intelligence and human workers interaction at team level: a conceptual assessment of the challenges and potential HRM strategies. *International Journal of Manpower*.

[33] Wissemann A. K., Pit S. W., Serafin P., Gebhardt H. (2022). Strategic guidance and technological solutions for human resources management to sustain an aging workforce: review of international standards, research, and use cases. *JMIR Hum Factors*.

[34] Chang P. C., Zhang W., Cai Q., Guo H. (2024). Does AI-driven technostress promote or hinder employees’ artificial intelligence adoption intention? a moderated mediation model of affective reactions and technical self-efficacy. *Psychology Research and Behavior Management*.

[35] Anwar R. S., Channa K. A., Shah S. M. M. (2021). Scope of combining the research methods in human resource management (HRM) and organizational behavior (OB). *Indian Journal of Economics and Business*.

[36] Saha E., Rathore P. (2024). The impact of healthcare 4.0 technologies on healthcare supply chain performance: extending the organizational information processing theory. *Technological Forecasting and Social Change*.

[37] Popa I., Cioc M. M., Breazu A., Popa C. F. (2024). Identifying sufficient and necessary competencies in the effective use of artificial intelligence technologies. *Amfiteatru Economic*.

[38] Kulkov I., Kulkova J., Leone D., Rohrbeck R., Menvielle L. (2023). Stand-alone or run together: artificial intelligence as an enabler for other technologies. *International Journal of Entrepreneurial Behavior and Research*.

[39] Yu X., Xu S., Ashton M. (2023). Antecedents and outcomes of artificial intelligence adoption and application in the workplace: the socio-technical system theory perspective. *Information Technology and People*.

[40] Rizany I., Hariyati R. T. S., Handayani H. (2018). Factors that affect the development of nurses’ competencies: a systematic review. *Enfermería Clínica*.

[41] Pai R. Y., Shetty A., Shetty A. D. (2022). Integrating artificial intelligence for knowledge management systems–synergy among people and technology: a systematic review of the evidence. *Economic Research-Ekonomska Istraživanja*.

[42] Qamar F., Afshan G., Rana S. A. (2023). Sustainable HRM and well-being: systematic review and future research agenda. *Management Review Q*.

[43] Demerouti E., Bakker A. B., Nachreiner F., Schaufeli W. B. (2001). The job demands-resources model of burnout. *Journal of Applied Psychology*.

[44] Li P., Bastone A., Mohamad T. A., Schiavone F. (2023). How does artificial intelligence impact human resources performance, evidence from a healthcare institution in the United Arab Emirates. *Journal of Innovation and Knowledge*.

[45] Dicuonzo G., Donofrio F., Fusco A., Shini M. (2023). Healthcare system: moving forward with artificial intelligence. *Technovation*.

[46] Silva A. (2023). The impact of technological advancements on HR practices and leadership in Brazil. *Journal of Human Resource and Leadership*.

[47] Alanazi A. T. (2022). Digital leadership: attributes of modern healthcare leaders. *Cureus*.

[48] Hassani H., Silva E. S., Unger S., TajMazinani M., Mac Feely S. (2020). Artificial intelligence (AI) or intelligence augmentation (IA): what is the future?. *AIDS*.

[49] Parasuraman A., Alenezi A. (2000). Technology readiness index (TRI) a multiple-item scale to measure readiness to embrace new technologies. *Journal of Service Research*.

[50] Karaca O., Çalışkan S., Demir K. (2021). Medical artificial intelligence readiness scale for medical students (MAIRS-MS)–development, validity and reliability study. *BMC Medical Education*.

[51] Sunmoo Y., Yen P. Y., Bakken S. (2009). Psychometric properties of the self-assessment of nursing informatics competencies scale. *Studies in Health Technology and Informatics*.

[52] Carless S. A., Wearing A. J., Mann L. (2000). A short measure of transformational leadership. *Journal of Business and Psychology*.

[53] Lutwama G. W. (2011). *The Performance of Health Workers in Decentralised Services in Uganda*.

[54] Al-Dakroury W. A., Alnemary F. M., Alnemary F. (2022). Autism in the Kingdom of Saudi Arabia: current situation and future perspectives for services and research. *Perspectives of the ASHA Special Interest Groups*.

